# Spark-Based Parallel Genetic Algorithm for Simulating a Solution of Optimal Deployment of an Underwater Sensor Network

**DOI:** 10.3390/s19122717

**Published:** 2019-06-17

**Authors:** Peng Liu, Shuai Ye, Can Wang, Zongwei Zhu

**Affiliations:** 1 National and Local Joint Engineering Laboratory of Internet Application Technology of Mines, Xuzhou 221008, China; liupeng@cumt.edu.cn; 2 Internet of Things Perception Mine Research Center, China University of Mining and Technology, Xuzhou 221008, China; 3 School of Information and Control Engineering, China University of Mining and Technology, Xuzhou 221116, China; TS16060253P3@cumt.edu.cn; 4 Aerospace Products Division, East China Institute of Computing Technology, Shanghai 201808, China; wangcan003457@ecict.com.cn; 5 Suzhou Institute of University of Science and Technology of China, Suzhou 215123, China

**Keywords:** genetic algorithm, multi-peak function, underwater sensor network, parallel computing, large-scale data, Spark, Hadoop

## Abstract

Underwater sensor networks have wide application prospects, but the large-scale sensing node deployment is severely hindered by problems like energy constraints, long delays, local disconnections, and heavy energy consumption. These problems can be solved effectively by optimizing sensing node deployment with a genetic algorithm. However, the genetic algorithm (GA) needs many iterations in solving the best location of underwater sensor deployment, which results in long running time delays and limited practical application when dealing with large-scale data. The classical parallel framework Hadoop can improve the GA running efficiency to some extent while the state-of-the-art parallel framework Spark can release much more parallel potential of GA by realizing parallel crossover, mutation, and other operations on each computing node. Giving full allowance for the working environment of the underwater sensor network and the characteristics of sensors, this paper proposes a Spark-based parallel GA to calculate the extremum of the Shubert multi-peak function, through which the optimal deployment of the underwater sensor network can be obtained. Experimental results show that while faced with a large-scale underwater sensor network, compared with single node and Hadoop framework, the Spark-based implementation not only significantly reduces the running time but also effectively avoids the problem of premature convergence because of its powerful randomness.

## 1. Introduction

An underwater wireless sensor network (UWSN) refers to a network composed of sensors deployed in the designated water. These sensors, featured with low energy consumption and limited communication distance, constitute a network automatically by means of their self-organizing ability. Then the ad hoc UWSN will collect, process and upload data of the designated water to servers [1]. Due to its wide and crucial application in marine resource exploration, marine environmental monitoring, and marine military field, UWSN has aroused wide concerns among industrial, academic, and military fields [2]. A UWSN is different from conventional wireless sensor networks (WSN) mainly in that it is used to conduct three-dimensional detection of a water environment. The underwater environment is complex and changeable, which makes fast-fading acoustic signals the only available means to transmit data. However, this definitely pushes up the sensor cost and network energy consumption, and makes many conventional WSN methods unsuitable for UWSN. Therefore, many scholars have conducted studies on UWSNs, to be specific, on topology control protocols, location algorithm, node deployment, coverage control, etc. [2,3,4].

Deploying UWSN nodes reasonably can save sensor resources, improve network efficiency, balance network energy consumption, and prolong network lifetime. Accordingly, the strategy of node deployment has always been a hot research topic of UWSNs [5]. Gupta [6] established a 2D-WSN mathematical model for node deployment and conducted numerical simulation by genetic algorithm (GA). However, the model was not built in a three-dimensional environment and, thus, could not be applied to UWSNs directly. In addition, given that network holes often occur in the topological structure of UWSNs due to water currents or node failures, the node locations need adjusting from time to time, which, in turn, increases the difficulty of sensor network deployment. Some research [7,8] shows that the node deployment of UWSNs is more complex than that of a 2D-WSN; meanwhile, the computation of the former is much heavier than that of the latter. Therefore, in the case of the massive deployment of a UWSN, the traditional GA is far from competent when handling these problems due to its low capability, long running time, and poor solution quality. By contrast, a parallel genetic algorithm shows a remarkable competitive edge. Parallel GAs [9,10,11] divide the evolution population into many splits and assign them to different nodes of a computing cluster, respectively, thus achieving a high degree of parallel computation and meeting the timeliness demand of the UWSN when deployed in a real underwater environment.

Hadoop [12] is a distributed computing framework developed by Google to deal with large-scale data service. Its kernel modules include MapReduce and the Hadoop Distributed File System (HDFS). Essentially, MapReduce is a distributed programming model to handle big data on large-scale computer clusters. Compared with conventional parallel programming models, such as massively parallel processing (MPP), MapReduce achieves elegant abstraction in the bottom layer, which enables users to develop distributed applications easily. As for the other kernel module, HDFS realizes the distributed reliable storage and access of mass data by various strategies, including storing redundant copies, haul storage, etc. Hadoop clusters can be employed to handle complex distributed programs, like GA, to achieve high parallelization. Through the full display of its scale effect, high-speed computation and storage can be conducted, which can sensibly improve the computational efficiency of GA. However, when complicated iterative operations are conducted with MapReduce, continuous disk reads and writes between operations consume a large amount of time, thus leaving much space for further improvement on computational efficiency [13].

Based on memory computing, the state-of-art distributed computing framework Spark [14] supports complex query, stream computing, and classified data mining of large-scale datasets, which turns lightweight quick processing into a reality. Memory computing has also been used in many areas of edge computing [15,16,17,18,19], and we have carried out a series of studies on memory power [20,21,22]. Owing to the unique computation mechanism of resilient distributed datasets (RDD) of Spark, the intermediate results of every iteration can be stored in the internal memory for the next iteration, which, in turn, remarkably improves the multiple iteration efficiency of GA.

The major contribution of this paper is summarized as follows:

(1) The Shubert multi-peak function (SMPF) is used to simulate deployment of underwater sensor network. By calculating the extremums of the Shubert multi-peak function (ESMPF), the simulating optimal deployment sites can be obtained.
(2) Based on RDD computation model of the Spark framework, a parallel GA for optimizing the deployment of a UWSN (DUWSN) is designed and implemented.
(3) By the comparison with the GAs based on single-node and Hadoop, it is verified that the proposed GA runs more efficiently while showing a higher accuracy.

The rest of this article is arranged as follows: Section 2 gives the system application model. Section 3 introduces the application of single-node genetic algorithm in optimizing DUWSN. Section 4 describes the Hadoop-based parallel GA for optimizing DUWSN. Section 5 presents the proposed Spark-based parallel method. Section 6 offers the experiment description. Part 7 delivers the conclusion. 

## 2. System Application Model

**Figure 1:**
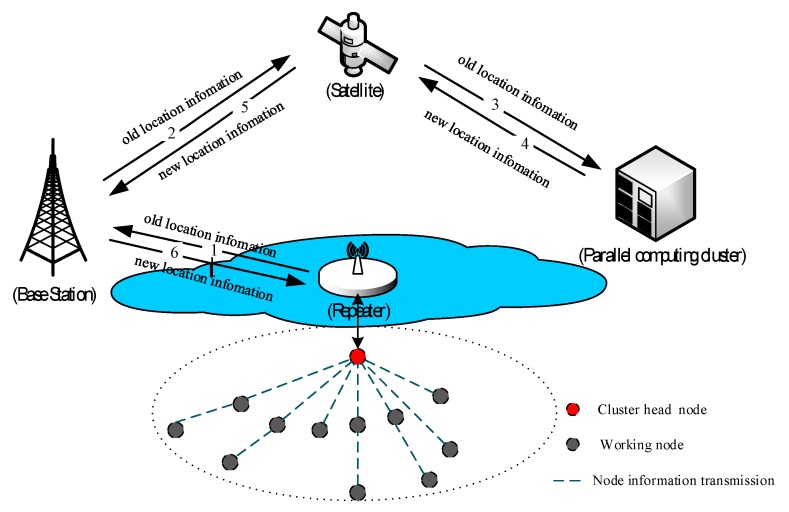
The process framework of location updates of an UWSN.

The process framework of location update of UWSN is shown as Figure 1. At certain regular intervals (e.g., 30 min), the working nodes transmit information of their location and residual energy to the cluster head node. Judging by the two criteria (1. Residual energy is sufficient; 2. heartbeat signal is normal (no disconnection or noticeable delay)), the cluster head selects the qualified working nodes and sends their location to the parallel computing cluster on the ground, through the communication link of a repeater, a base-station, and a satellite. Then, the parallel optimization computation is immediately conducted by the computing cluster, and the result (i.e., the optimal deployment locations) is transmitted back to the cluster head by the same way of a satellite, a base station, and a repeater. Finally, the cluster head distributes the new deployment locations to every working node and the latter adjusts its location to the optimal deployment accordingly. 

It should be noted that the cluster head node, as the forwarding and controlling node of the whole cluster, undertakes a heavy workload in the topological structure of UWSN as shown in Figure 1. To ensure the sufficient energy of the cluster head, a cluster head reselection mechanism is introduced which can balance the energy consumption of the whole network and guarantee the normal function of the cluster head, thus prolonging the overall lifetime of the network. 

As is presented in Section 1, in view of the complex underwater environment, the traditional genetic algorithm has been abandoned gradually due to its poor computation efficiency and accuracy. The new-generation parallel optimization has become a popular research direction for the fast and accurate deployment of UWSNs. Therefore, a Spark-based parallel genetic algorithm is proposed in this paper, aiming at optimizing UWSN deployment efficiently and accurately. To be specific, the Shubert function is used to simulate the UWSN deployment that is mapped into corresponding coordinate figures in the function. Then a Spark-based parallel genetic algorithm is employed to obtain the optimal results. A specific description is shown in the following sections.

## 3. Single-Node GA for Solving ESMPF

Generally, the mathematical model of single-node GA can be expressed according to Equation (1), where *C* denotes the encoding scheme of chromosomes, *P* stands for initial population, *F* denotes the Shubert fitness function, *N* stands for the size of population, Θ stands for the selection operator, Γ denotes the crossover operator, Ψ denotes the mutation operator, and *T* stands for the termination condition for the algorithm. As is well known, GA is a somewhat globally heuristic optimization algorithm based on the evaluation of chromosome fitness, which actually means the use of existing data to guide the optimization search direction can significantly improve the algorithm quality finally:

(1) SGA=(C,P,F,N,Θ,Γ,Ψ,T)

### 3.1. Shubert Multi-Peak Function

**Figure 2:**
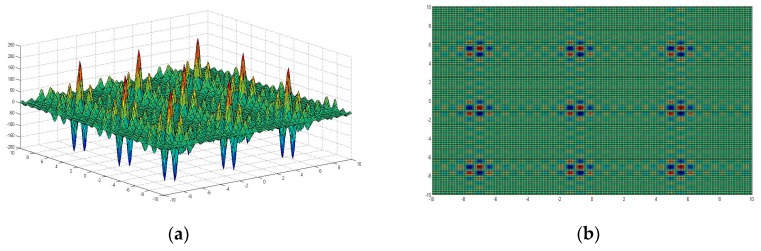
(**a**) Shubert function simulation view 1 (along X-axis and Y-axis); and (**b**) Shubert function simulation view 2 (along Z-axis).

Given that the UWSN environment is complex and changeable, to facilitate the study of DUWSN, the target area is assumed to be a cuboid in this article. By the simulation of the Shubert function model [23], the extremums of the Shubert multi-peak function (ESMPF) can be obtained, which can be then used to locate the optimal deployment sites of the UWSN. This can be justified by the fact that the Shubert objective function has multi-dimensional parameters that can simulate the complex underwater environment effectively, and its optimized objective can reflect vividly the deployment objective of the UWSN. In addition, as can be seen from Figure 2, the distribution of ESMPF itself constitutes a 3D space that shares a natural similarity with the distribution of underwater sensing nodes. Therefore, the calculation of ESMPF is used to simulate the optimal deployment strategy of underwater sensing nodes in this paper. Shubert function [24], which is set as the fitness function in this paper, is a typical multi-peak function (function with multiple peak values). According to our visualization analysis, the Shubert function has 760 local extremums and 18 global extremums within the interval between −10 and 10, as shown in Figure 2. The Shubert function formula is as follows:

(2) $$f(x,y)=\sum_{i=1}^{5}i\cos[(i+1)x+i]\times \sum_{i=1}^{5}i\cos[(i+1)y+i]\;-10<x<10,-10<y<10$$

In actual applications, it is more often that the global extremums and alternative local extremums are needed for the function with multiple peak values. However, the traditional algorithm for solving extremums is generally denounced for its low efficiency and poor accuracy. Meanwhile, it often easily falls into the dilemma of local optimal values. 

### 3.2. The GA Dataset Encoding for SMPF

The design of individual encoding scheme, as the first step of genetic algorithm, is of vital importance. When GA is used to solve the Shubert function, the decision parameters are not to be operated on directly. Instead, the feasible solution set of the objective function is mapped into the search space of the algorithm, which is called “individual encoding”. Then the GA is to be applied to the encoded individuals. 

Being quite easy, practicable for GA operations, and consistent with the principle of minimum symbol set encoding, binary coding is adopted as the encoding scheme of the Shubert function. According to the methodology of binary coding, the feasible solutions of the function are converted into chromosome strings that are composed of 0 and 1. The total number of binary coding strings is related with the accuracy of function solutions ϕ. *X*_max_ and *X*_min_ denote the maximum value and minimum value of horizontal ordinate in the space. The formula of accuracy ϕ is as follows:

(3) $$\phi =\frac{X_{\max}-X_{\min}}{2^{l}}$$

To achieve higher and higher accuracy of function parameters, the corresponding binary strings have to be longer and longer. However, this inevitably leads to the sharp expansion of search space and the increase of calculation, which in turn reduces the search efficiency of the GA. After considering the efficiency of the frameworks involved and weighing the relation between accuracy and calculation amount, this paper uses nine-digit binary coding to denote the two decision parameters “x” and “y”. The nine-digit binary coding string can represent 512 figures between 0 and 511, thus discretizing the definition domain of “x” and “y” into 511 equal intervals with 512 discrete points (two endpoints included). Suppose that the discrete points from −10 to 10 represent the binary codes between 000000000(0) and 111111111(511) correspondingly, then the mapping relation is expressed as follows:

(4) $$\begin{array}{l} 000000000=0\;\to X_{\min}\\ 000000001=1\;\to X_{\min}+\varphi \\ 000000010=2\;\to X_{\min}+2\varphi \\ \vdots \;\vdots \;\vdots \\ 111111111=2^{9}-1\to X_{\max} \end{array}$$

Then two nine-digit binary coding strings are used to represent “x” and “y”. When the two strings are combined together to form an eighteen-digit binary coding string, it is the chromosome encoding for the Shubert function. By means of this encoding scheme, 262,144 pairs of chromosome samples can be produced in the whole solution space, which constitutes the sample database of the Shubert function. Meanwhile, there exists the corresponding one-one mapping relation between he coordinate solution space and the GA search space. According to the accuracy *φ*, this scheme can guarantee that a large amount of homogenous discrete points is produced and uniformly distributed in the solution space. This, to some degree, can ensure both the solutions accuracy and the search capability of genetic space. 

When the genetic evolution is finished, the last generation of population set needs to be decoded to obtain the results. The decoding function for the binary coding string $X=b_{l}b_{l-1}\cdots b_{2}b_{1}$ can be expressed as follows:

(5) $$x,y=X_{\min}+\left(\sum_{1}^{l}b_{i}\cdot 2^{i-1}\right)\cdot \frac{X_{\max}-X_{\min}}{2^{l}-1}\;,\;l=9$$

### 3.3. Single-Node Implementation

**Figure 3:**
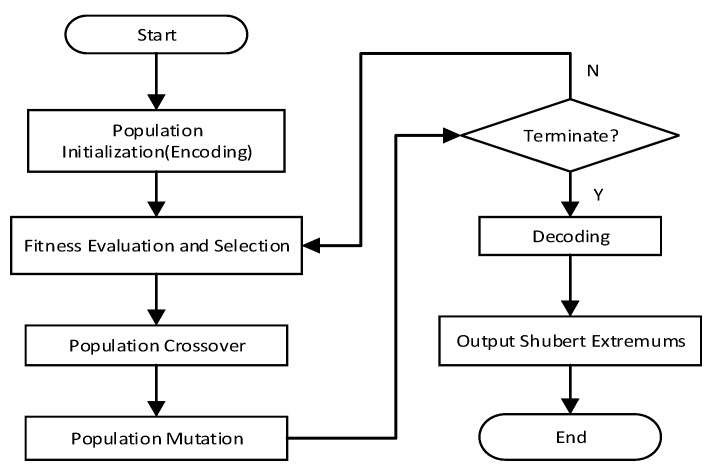
Single-node GA for solving ESMPF.

GA is a kind of searching strategy for global optimization based on genetic evolution. Compared with other kinds of algorithms, GA is more suitable for DUWSN. The process of single-node GA for solving ESMPF (GAESMPF) is shown in Figure 3.

1. Population Initialization: To input 262,144 pairs of binary coding string into the sample database of Shubert function and set them as the initial population.
2. Fitness Evaluation: Given that the individual fitness should be non-negative, a linear non-negative conversion should be done to the Shubert function and the fitness corresponding to each individual should be calculated.
3. Selection Operator: According to the individual fitness and selection probability, the next generation can be selected out by means of roulette.
4. Crossover Operator: All the individuals of the population should be coupled in a random and pairwise way, and single-point crossover should be conducted according to the crossover probability.
5. Mutation Operator: To traverse through each gene locus of every individual and reverse the loci which satisfy the mutation probability.
6. Decoding and Calculation: When the termination conditions are met, the individuals of the last generation are decoded with functions to obtain the corresponding function-value set and then the values are sorted to locate the extremums.

Careful study of Figure 3 can reveal the deficiency of single-node GAESMPF. Since it is a kind of serial operation, all the crossover, mutation and selection operators are conducted within the single population. Thus, the individuals located around the local extremums possess high probability to be selected. Consequently, the sample individuals would be more inclined to center around the first-solved local extremum after several times of iteration, which, actually, severely limits the further search space. In addition, in view of the changing underwater environment, the computation time of single-node GA is too long, which means that it is more likely that the optimal deployment location obtained may have become useless and unsuitable for the real-time underwater environment [25]. Contrarily, if the whole population is divided into many splits, each of which can conduct parallel searching under the guidance of different compute nodes. This can greatly reduce the optimization time. Meanwhile, samples can be taken from the individuals near every local extremum in each split, thus greatly enlarging the space for further searches. In other words, parallel searches by divided splits can retain the samples near different local extremums to avoid premature convergence to some degree. Thus far, although numerous improvement methods for single-node GA have emerged, the parallel GAs still boast of incomparable advantage in terms of efficiency and accuracy in UWSN deployment [26]. 

## 4. Hadoop-Based Parallel GAESMPF

**Figure 4:**
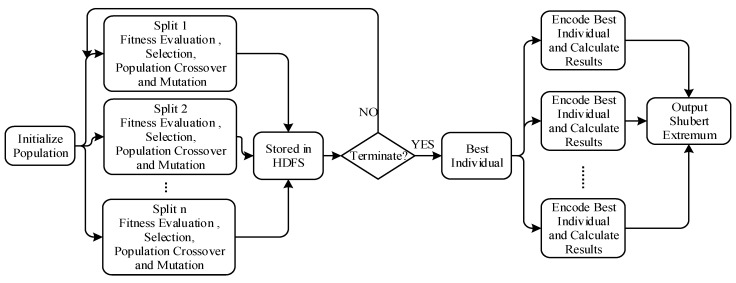
Hadoop-based parallel GAESMPF.

In order to set a benchmark for the proposed Spark-based parallel GAESMPF, Hadoop-based parallel GAESMPF is designed and implemented here, whose core concept is to convert the single-node serial iterations into the corresponding MapReduce parallel operations [27]. The actual process is shown in Figure 4.

### 4.1. Process of Parallel Genetic Iteration

To begin with, we input Shubert sample code set into HDFS. Then we read the pre-stored code set from HDFS as the individuals of the initial population and divide them into a certain number of splits. Next, we calculate the Shubert function values of individuals in every split and set the values as the individual fitness. Then we take the key-value pair <individuals, fitness> as the input and establish independent MapReduce tasks in every split. Finally, we conduct selection, crossover, and mutation, and store the evolved individual key-value pair <individuals, fitness> into HDFS [28]. The pseudo code of genetic process is as Algorithm 1.

**Algorithm 1.** Parallel genetic iteration.**Input:** N: Shubert sample code set    T: maximum times of iteration **Output:** the individual encoding key-value pair of the last generation 
1.To read N from HDFS2.To initialize the population3.To assign the initial population to different nodes4.For every individual i ∈ Initial population do5.To calculate the fitness of every individual6.To map them into key-value pair <individuals, fitness>7.End for8.Sort the individuals by fitness9.t = 010.while (t < T)14.{12.For population/2 times do13.To select the parent individuals randomly from the population F1, F214.crossover (F1, F2)15.{determining crossover-factor f1, f2}

16.crossover individuals:17.{18.F1’ = F1&f1|F2&f219.F2’ = F1&~f1|F2&~f220.}21.mutation (F1’, F2’)22.{determining mutation-factor f023.mutation individuals F” = F’^f024.}25.To select excellent individuals26.End for27.t = t + 128.}29.Output the individual set of the last generation (best Individuals)30.End


### 4.2. Process of Searching Extremums

During the process of searching extremums, the main task is to judge the termination conditions and search the extremums of the population. When the termination conditions are met, the population is decoded to obtain Shubert function values with a sorted list. Then the global extremums of the Shubert function and its corresponding variables are output. The pseudo-code of optimizing process is as Algorithm 2. 

**Algorithm 2.** Searching extremums.**Input:** N: Shubert sample code set of the last generation**Output:** the extremums of Shubert function
1.To read N from HDFS2.To assign the last generation population to different nodes3.For every individual i ∈ the individuals of the last generation do4.To decode the individuals5.To calculate the extremum of Shubert 

6.To map the function value and corresponding variable into key-value pair <value, (x, y)>7.End for8.Sort the function values by key9.To output the extremums of Shubert function and corresponding variable10.End


In the process of Hadoop-based parallel genetic iteration, the default operation of the Reduce function will sort the evolved population and override the storage with the default text name. This operation disrupts the previous order of the population, thus sharply increasing the randomness and expanding the search space during the next iteration. Compared with single-node GAESMPF, the advantage of the Hadoop-based scheme lies in the fact that it can avoid local optimization and improve the accuracy of solutions to some degree. Unfortunately, in terms of operation efficiency of the deployment of a UWSN, the Hadoop-based scheme still falls short.

## 5. Spark-Based Parallel GAESMPF

**Figure 5:**
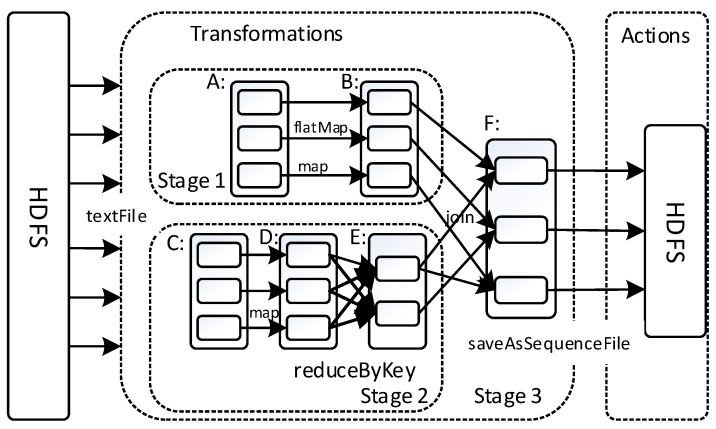
Spark operating mechanism.

As is well known, Hadoop MapReduce is not equipped with a memory operation mechanism [29]. Therefore, the population data needs to be read from disk every time an iteration is conducted, thus greatly increasing the running time and failing to utilize fully the parallel potential of the GA. Different from the Hadoop-based implementation, the parallel design of the Spark-based GAESMPF sets its ground upon RDD (resilient distributed dataset) which is unique to Spark. Briefly speaking, RDD is a resilient distributed storage system with high fault-tolerance. It stores large-scale data in the local memory of different computing nodes and conducts memory-based parallel computation in distributed clusters [30]. Generally, the operations of RDD fall into two categories: (1) Transformation, and (2) action. In short, the former is to create a new RDD based on an existing RDD, and the latter is to conduct actual parallel computation on RDD and then send back a displayable or storable result to the master control program. It is noteworthy that the transformations of RDD belong to *lazy* execution, which means that the transformations accumulated are not triggered in turn until the next action comes up (as shown in Figure 5).

In order to compare with single-node GAESMPF and Hadoop-based parallel GAESMPF, the Shubert code set is read from HDFS to conduct the genetic operation. To obtain the optimal solution, iterations need to be done continually. Whenever iterations are done, GA updates the code information according to the split results. The updated splits serve as the key factor for the ergodic selection during the next iteration. Therefore, when the current iteration is finished, the Spark-based GAESMPF, by the mechanism of utilizing the memory buffer of RDD, stores the updated split information in the memory of each node as intermediate data for the next iteration [31,32]. 

During the iterations of GA, the evolutionary process of splits in each node is completely independent. Accordingly, the Spark-based GAESMPF divides itself into partitions in line with the number of nodes. The partitions are then assigned to each node to create RDDs parallel to each other. For every RDD (which stands for the split in each node), the operation mode is parallel. After the operations of selection, crossover, and mutation are conducted by the RDD mechanism on Spark, the parallel execution of the whole population evolution is completed. 

**Figure 6:**
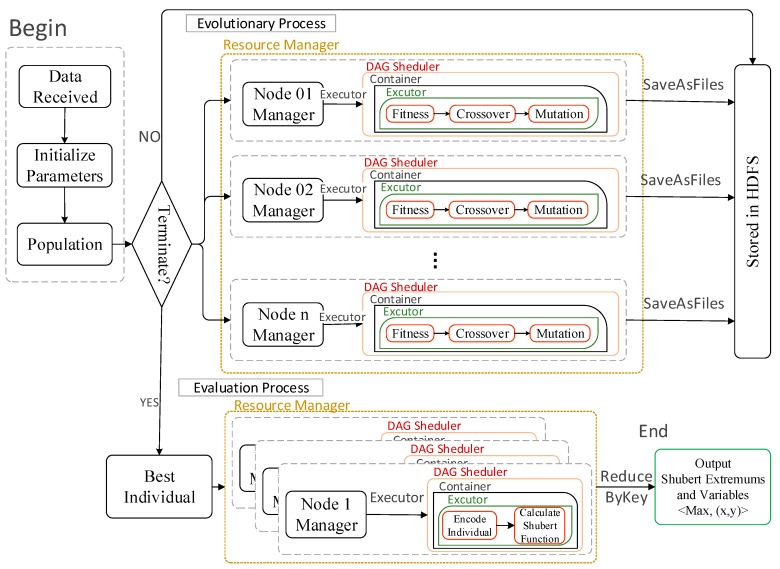
Spark-based parallel GAESMPF.

According to the above analysis, the Spark programming model is used to realize the parallel implementation of GA for multi-peak function extremums. The actual process of Spark-based GAESMPF is shown as Figure 6.

### 5.1. Phase of Parallel Genetic Operation

The genetic operation phase consists of four operations (fitness calculation, selection, crossover, and mutation) which can be conducted with RDD model on Spark framework. The high compatibility between Spark and HDFS makes it possible to deal with the text data stored in HDFS line-by-line. Therefore, the initial population can be stored as individual samples in HDFS line-by-line.

**Fitness calculation:** Firstly, read the code set of Shubert feasible solutions (total data employed in this paper are 262,114 pieces) from HDFS as the initial population of the GA. Then distribute the initial population to each node evenly and calculate the individual fitness with Shubert function on each node. Next *map* the key-value pair of individual code and fitness and store it in the new RDD in the form of *<individuals, fitness>*. In this paper, *x* and *y* denote the location information of working nodes. The fitness formula is the Schubert function that goes as follows:

(6) $$f(x,y)=\sum_{i=1}^{5}i\cos[(i+1)x+i]\times \sum_{i=1}^{5}i\cos[(i+1)y+i]$$

**Selection:** Natural selection refers to a category of selection strategies for future generations. Common strategies include roulette, tournaments, and elite retention. Roulette is to extract progeny with a certain probability, repeated n times, and the probability of each individual being drawn is as follows:

(7) $$p_{i}=\frac{f\left(x_{i},y_{i}\right)}{\sum_{j=1}^{n}f\left(x_{j},y_{j}\right)}$$

Conduct natural selection upon every individual in RDD on each node, then traverse the characteristics of all the key-value pairs in combination with *Map*. In every selection process, employ a fitness value as the criterion to select out the individuals that meet the requirements for the next generation. Finally, create the RDD of the new population based on “the survival of the fittest” and store it in the memory. To avoid local optimization and increase the individual randomness, the individual data on each node is re-sorted for the next operation.

**Figure 7:**
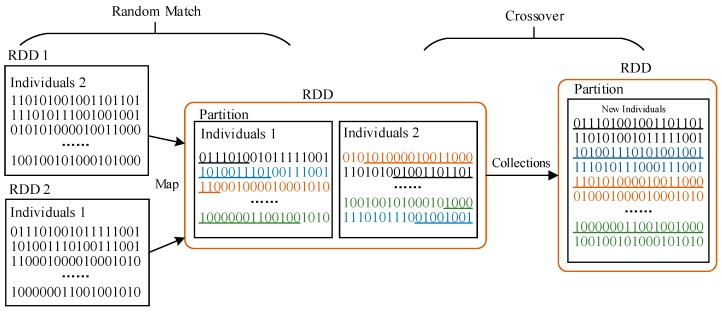
Illustration of crossover.

**Crossover:** Firstly, sample all the individuals of RDD on each node by means of the *take* function and store them evenly in two lists. Then create two RDDs out of the two lists by means of the *parallelize* function and form key-value pairs to realize the random match of two individuals of the parent generation. Next use the *Map* function to conduct single-point crossover operation upon the key-value pairs (*<individual, individual>*) line-by-line. Then generate the corresponding crossover-factor $f_{1}(x)$ and $f_{2}(x)$, retrieve the key-value pair after crossover operation and create the population RDD by means of the *Map* function according to Equations (9) and (10). Finally, the newly created RDD is stored in the memory. The crossover process is illustrated in Figure 7.

(8) $$f_{1}(x)=1111111|00000000000\;0,f_{2}(x)=0000000|1111111111\;1$$

(9) $$f_{a}’(x)=f(x)\&f_{1}(x)|f(x)\&f_{2}(x)$$

(10) $$f_{b}’(x)=f(x)\&\sim f_{1}(x)|f(x)\&\sim f_{2}(x)$$

**Figure 8:**
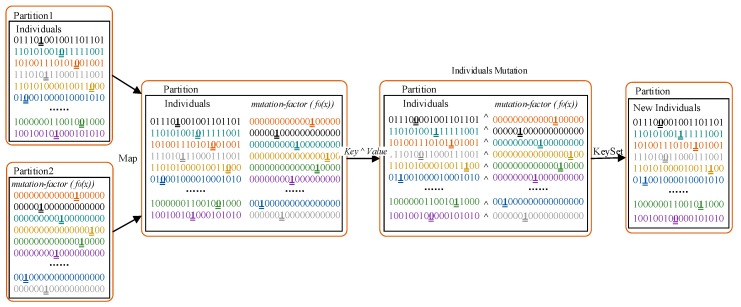
Illustration of mutation.

**Mutation:** after crossover, use *Map* to read every individual in the RDD line-by-line and traverse all the gene loci of every individual code. The random number generated can be used as the criterion to assess if the individual code has met the mutation condition, then generate the corresponding mutation-factor $f_{0}(x)$. For the loci that meet the mutation condition, a negation operation is conducted, thus creating a new individual as shown in the Equation (12); otherwise, the individual code should be output directly. After mutation, the population RDD created is stored in the memory. The mutation process is illustrated in Figure 8.

(11) $$f_{0}(x)=0000\underline{1}0000000000000$$

(12) $$f”(x)=f’(x)\wedge f_{0}(x)$$

### 5.2. Phase of Searching Extremums

**Figure 9:**
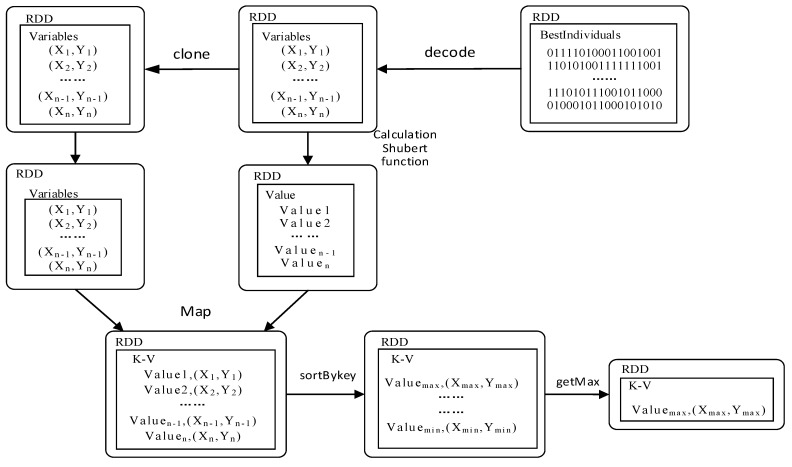
Searching optimal extremums for SMPF based on RDD.

Figure 9 displays the process of searching optimal extremums of the last-generation population. If the iteration times meet the preset termination condition, the RDD created in the end will be handled with the *Map* function. During the *Map* operation, the variable-decoding step is conducted to obtain Shubert function values. The extremums obtained and their corresponding variables are stored in the form of key-value pairs which, to be specific, is denoted as <Value, (X, Y)>. The key-value pairs are ranked by sortByKey and stored in HDFS. The result stored in HDFS is actually the collection of all node locations ranked by merits. In other words, the result includes not only the information of optimal node locations but also that of other node locations. This “whole result” can better meet the actual demand of optimization deployment of UWSN. The last step is to output the extremums of Shubert function and its corresponding variables. Thus, the corresponding variables (Xi, Yi) are the best locations for deployment of a UWSN.

## 6. Experiment and Analysis

The goal of the experiment is to verify the advantages of the proposed method, GAESMPF, especially for large-scale data, from the following two aspects:

1. High efficiency: Through the comparison of running time, speedup ratio, and average time consumed for iterations on single-node, Hadoop, and Spark, respectively, the high efficiency of the Spark-based GAESMPF is verified.
2. Accuracy and stability: Through comparison of the Shubert function extremums obtained with different times of iterations, the accuracy and stability of parallel GAESMPF based on Spark is verified.

### 6.1. Experiment Configuration

The computing cluster consists of 20 nodes (one master and 19 slaves). The same sample dataset is used on all the frameworks. The actual configuration and operation parameters are shown in Table 1. 

### 6.2. General Running Efficiency

The experiments use 262,144 pairs of chromosomes provided by the code database of Shubert function feasible solutions that has been established in previous sections. The iteration times are set at 30, 50, 70, 90, 110, 130, and 150, respectively. The experiment results are shown in Table 2. For every result of a row in Table 2, we take the average value of ten repeats of GAESMPF computation. 

**Figure 10:**
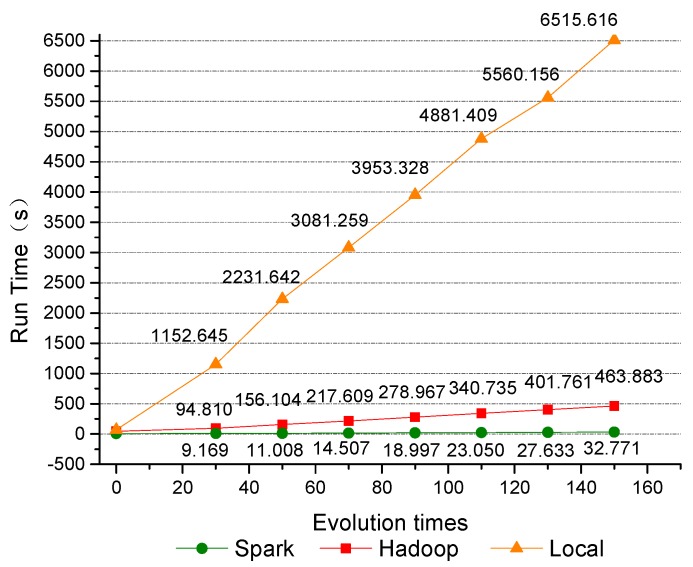
Running efficiency comparison of GAESMPF.

As can be seen from Table 2, compared with GAESMPF based on Hadoop or Spark, the single-node GAESMPF needs many more iteration times to reach the optimal extremum and, thus, needs a much longer running time. Compared with the single-node GAESMPF, the Hadoop-based GAESMPF boasts a much higher efficiency. Meanwhile, as is shown in Figure 10, the X-Y variations of iteration times and running time on the three frameworks are generally distributed linearly. As for the two parallel algorithms, Spark-based GAESMPF virtually saves ten times the running time compared with Hadoop-based GAESMPF. Moreover, the tendency of running time curves indicates that, as the iteration times increase, the running efficiency of Spark-based GAESMPF rises more sharply and the gap between the two distributed GAESMPFs becomes wider and wider. When the iteration times reach 150, Spark-based GAESMPF can save up 92.93% of the running time. Therefore, when the computing capacity is sufficient, the Spark-based GAESMPF can fully utilize its advantage of memory computing to reduce the running time significantly and, thus, greatly improve the timeliness of UWSN deployment.

The implementation of GAESMPF based on Hadoop provides a feasible operation process for the storage and transmission of evolved populations on each computing node. In this process, one iteration needs a map phase and a reduce phase. The two phases inevitably involve massive data transmission that is quite time-consuming. By contrast, for Spark-based GAESMPF, the process of population evolution is conducted completely on RDD in the memory. The population of RDDs is distributed evenly on each node in the Spark cluster. In the process of iteration, the population searches the optimal solution through a series of RDD conversion. These techniques can release the great potential of memory computing of the Spark framework. Moreover, it can be easily seen from Figure 10 that as the iteration times increase, Spark-based GAESMPF saves more and more time than Hadoop-based GAESMPF.

### 6.3. Solution Accuracy with Evolution Times

**Figure 11:**
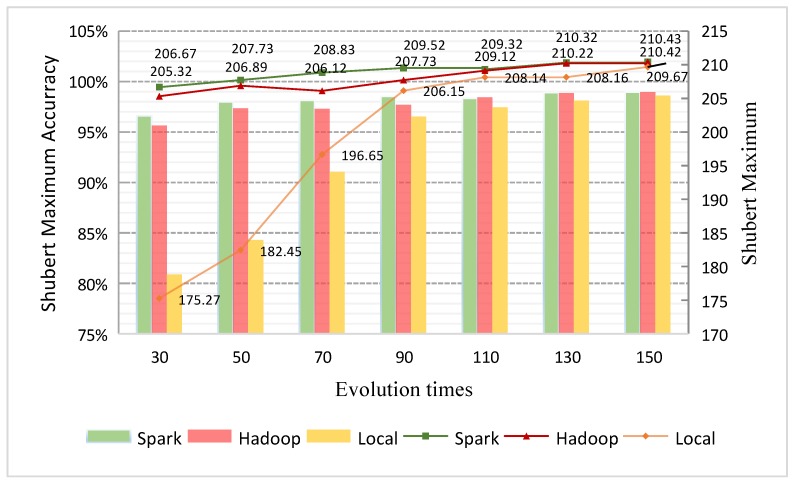
Solution accuracy comparison of GAESMPF.

In order to ensure the accuracy of extremums and minimize the influence of outliers, the average value of ten running results is taken as the analysis object. As can be seen clearly from Figure 11, as the times of iteration increase, the extremums of single-node GAESMPF keeps changing significantly. Moreover, compared with the results of two distributed versions, the error rate is relatively high and local extremums appear many times. Until the iteration times reach a certain degree (90 times), the accuracy of single-node GAESMPF tends to be stable. On the contrary, the accuracies of distributed versions fluctuate quite mildly and show no obvious correlation with the times of iteration. As is shown, the two distributed versions boast of relatively high accuracies even when the iteration times are quite small. In addition, as the iteration times increase, the results tend to approach the final exact extremums of the Shubert multi-model function. Therefore, the accuracy and stability of the distributed versions are remarkably higher than those of the single-node version.

It is noteworthy that Figure 11 demonstrates a vital characteristic of GAESMPF, that is, the high randomness brought forward by parallel computation. This characteristic makes it feasible to dig out the “genetic” potential to fully and effectively avoid local optimal solutions (LOS), which, in turn, remarkably improves the accuracy and stability of GAESMPF. Why do GAs easily fall into LOS? The main reason lies in the fact that in a single population the individuals near the LOS are more likely to be selected, which means the data samples in the genetic space will readily cluster around the LOS after several times of iteration. This will greatly limit the space for further searches. Thus, the purpose of dividing the initial population into multi-node splits is to ensure that samples are to be evenly distributed near every LOS. By doing so, the space for further searches will be expanded greatly and effectively. Therefore, based on the distributed computing framework, the GAESMPF conducts crossover and mutation within the splits on each node at first, and then integrates and stores the results. This will retain the samples around different LOS and, thus, significantly improve the search efficiency and accuracy.

### 6.4. Run Time of a Single Iteration

**Figure 12:**
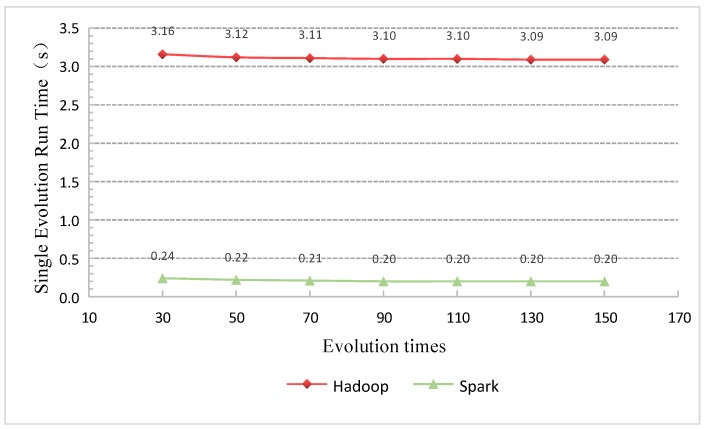
Comparison of the run time of a single iteration of GAESMPF.

The performance of GAESMPF can be further analyzed through the comparison of average run times for a single iteration. Figure 12 displays the run time distribution of single iteration at different evolution times on the two distributed frameworks. As the evolution times increase and exceed 80 times, the run time for a single iteration tends to be quite stable. The average run time for a single iteration on Hadoop is 3.09 s, while that on Spark is 0.22 s. In other words, the Spark-based version is 2.87 s faster than the Hadoop-based version when dealing with a single evolution. That is also to say that the working efficiency of the Spark-based version is improved by 13 times than the Hadoop-based version, thus ensuring the timeliness of UWSN deployment. 

### 6.5. Speedup Ratio Analysis

Experiments were conducted to evaluate the speedup ratio of GAESMPF based on Spark and Hadoop. The speedup ratio is a key indicator for the system performance under the same workload. For distributed versions, compared with the single-node version, the larger the speedup ratio is, the greater the working efficiency is. The formula of the speedup ratio is shown as follows:

(13) $$S_{p}=T_{1}/T_{p}$$

where $S_{p}$ denotes the speedup ratio, $T_{1}$ stands for the run time of GAESMPF based on a single node, and $T_{p}$ stands for the run time of GAESMPF based on distributed frameworks.

#### 6.5.1. Speedup Ratio with Different Evolution Times

**Figure 13:**
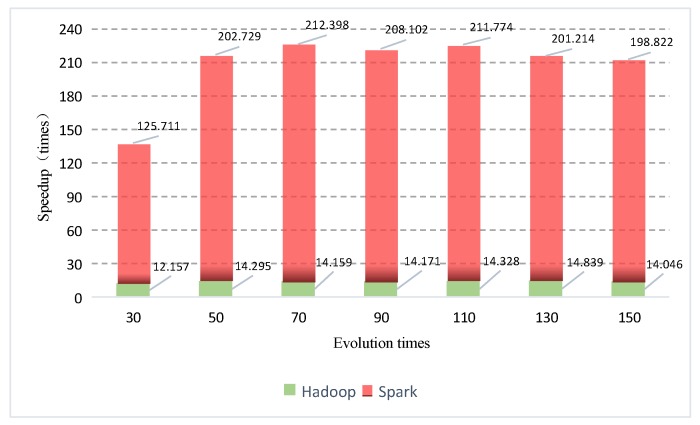
Comparison of the speedup ratio with different evolution times.

MapReduce is a batch-processing engine whose normal operation is to read data from the disc, deal with the data, and write the results back to the disc. Since Hadoop-based iterative evolution needs to read the disc constantly, it is quite time-consuming. On the Spark framework, intermediate data are stored in the memory rather than disc and similar operations are all conducted in memory, thus greatly improving the running efficiency. Figure 13 displays the speedup ratio distribution of Hadoop-based GAESMPF and Spark-based GAESMPF with different evolution times. As can be seen straightly, the average speedup ratio of the Spark-based GAESMPF is approximately 194.4 while that of the Hadoop version is about 13.9. This indicates that the Spark-based version possesses remarkable advantages over the Hadoop-based version in terms of the speedup ratio.

#### 6.5.2. Speedup Ratio with Different Node Numbers

**Figure 14:**
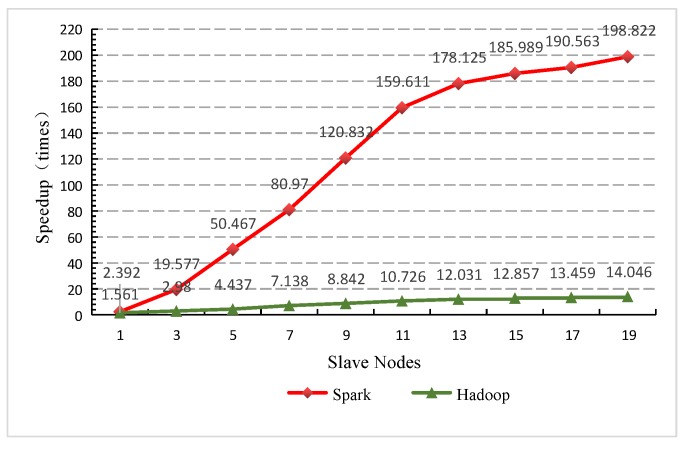
Comparison of speedup ratio with different node numbers.

As is designed in the experiment, when iterations reach 150 times, the speedup ratio is analyzed with different node numbers. From Figure 14 it can be seen that, as the node number increases, the gap of speedup ratio between the Spark version and Hadoop version becomes wider and wider. When the node number reaches the maximum 19, the speedup ratio of Spark version and Hadoop version is 198.822 and 14.046, respectively, the former 14 times greater the latter. This can be explained by the fact that, for time-consuming GAESMPF operations, as the number of working nodes increases, the Spark-based version occupies more and more obvious advantages over the Hadoop-based version in the process of the optimal deployment of the UWSN.

## 7. Conclusions

In this paper, a Spark-based parallel GA is proposed to simulate the optimal deployment of an underwater sensing network. Compared with single-node-based GA and Hadoop-based GA, the Spark-based parallel GA can significantly shorten the computation time of iterative evolution when dealing with large-scale underwater sensing nodes. As can be seen from the experiment results in Figure 10, the computation efficiency of the proposed method is improved by over 200 times compared with the single-node-based GA, and 15 times compared with the Hadoop-based GA. Thus, a remarkable improvement in the timeliness of solving the optimal deployment of a UWSN is achieved. Encouragingly, the innate natural randomness and distribution of the Spark-based parallel GA entitles it to avoid local optimization effectively, which further improves the accuracy of the proposed method and finally results in optimal deployment of the UWSN. Therefore, the proposed method of Spark-based parallel GA is rather promising in solving the optimized deployment strategy of a large-scale UWSN. In the future, the author will further investigate the deployment strategy and topology control protocols of a large-scale UWSN by means of the fusion of parallel GA and parallel machine learning algorithms. 

## Figures and Tables

**Table 1:** Configuration of the experiments.

Node	Maximum Population	Crossover Rate	Mutation Rate	CPU (Core)	Memory	Network	JDK Version	Hadoop Version	Spark Version
Master	262,144	0.9	0.05	4	6 GB	1 GB/s	1.7.0	1.2.1	1.6.0
Slave (1~19)	262,144	0.9	0.05	4	6 GB	1 GB/s	1.7.0	1.2.1	1.6.0

**Table 2:** The optimal result of GAESMPF based on single-node, Hadoop, and Spark.

Iterations	Single-Node		Hadoop		Spark	
	Time/s	Extremum	Time/s	Extremum	Time/s	Extremum
30	1152.645	175.27	94.810	205.32	9.169	206.67
50	2231.642	182.45	156.104	206.89	11.008	207.73
70	3081.259	196.65	217.609	206.12	14.507	208.83
90	3953.328	206.15	278.967	207.73	18.997	209.52
110	4881.409	208.14	340.735	209.12	23.050	209.32
130	5560.156	208.16	401.761	210.22	27.633	210.32
150	6515.616	209.67	463.883	210.42	32.771	210.43
